# Endovascular stenting for symptomatic atherosclerotic stenosis of the anterior and posterior cerebral arteries: a case series

**DOI:** 10.3389/fneur.2025.1700495

**Published:** 2026-01-13

**Authors:** Lei Wang, Zhiyong Zhang

**Affiliations:** 1 Department of Neurology, Beijing Geriatric Hospital, Beijing, China; 2 Neurovascular Intervention Center, Beijing Geriatric Hospital, Beijing, China; 3 Neurology and Neuroscience Center, Beijing Geriatric Hospital, Beijing, China

**Keywords:** anterior cerebral artery, endovascular stenting, intracranial atherosclerotic stenosis, ischemic stroke, posterior cerebral artery

## Abstract

**Background:**

Symptomatic intracranial atherosclerotic stenosis (sICAS) in the anterior cerebral artery (ACA) or posterior cerebral artery (PCA) may result in disability even with the best pharmacological intervention. There is a paucity of evidence regarding endovascular procedures for sICAS associated with ACA or PCA.

**Methods:**

From January 2022 to December 2024, 12 patients with medically refractory sICAS associated with ACA or PCA, who received percutaneous transluminal angioplasty and stenting (PTAS), were included for analysis. Exclusion criteria included (1) non-atherosclerotic stenosis, concomitant infarcts in other vascular territories, antiplatelet contraindication, a baseline modified Rankin Scale (mRS) score of 3 or above, and (2) concomitant disease with survival < 2 years. sICAS with 70–99% stenosis and 2 or more strokes in the same vascular territory, with at least 1 stroke occurring during medical therapy, is considered refractory to medical treatment. Clinical data from these patients were collected and analyzed.

**Results:**

In 12 patients, three stenotic lesions were in the A2 segment of the ACA, and 9 were in the P1 or P2 segments of the PCA (stenosis > 70%). All lesions were treated with PTAS (1 with the NOVA intracranial sirolimus-eluting stent system, 1 with the Neuroform EZ stent, and 10 with the Neuroform Atlas stents), achieving a 100% technical success rate (residual stenosis < 30%). Dissection with unrestricted flow occurred in 1 patient (1/12) during stent placement, which disappeared 12 months later. The follow-up time ranged from 3 to 18 months (≥ 6 months in 11 patients). In the follow-up, 1 patient suffered a recurrent stroke (8.3, 95% CI: 0.21–38.5%) due to the cessation of antiplatelet and anticoagulant medication 2 months after endovascular stenting; however, the remaining 11 patients exhibited a fair outcome with a modified Rankin Scale of 1 or 0 (91.67, 95% CI: 61.5–99.8%) and no in-stent stenosis (0, 95% CI: 0–26.5%).

**Conclusion:**

Endovascular stenting with newer self-expanding stents may be feasible and procedurally safe for patients with medically refractory sICAS associated with ACA or PCA. However, this therapy should not be treated as generalizable or low-risk. Further research is necessary for these patients.

## 1 Introduction

Intracranial atherosclerotic stenosis (ICAS) is a leading cause of ischemic stroke worldwide (1). The risk of stroke recurrence, even with aggressive medical management, reaches 20% within a year for patients with ICAS and up to 37% for those with symptomatic ICAS (sICAS) and border zone infarction or inadequate collateral circulation (2–4). Intrinsic atherosclerosis is the primary cause of anterior cerebral artery (ACA) and posterior cerebral artery (PCA) infarction in the East Asian region (5–7). The symptoms in patients with ACA and PCA infarction can be significantly disabling, including motor dysfunction, extrapyramidal symptoms, altered mental status, and visual field defects (8–10). In a large population-based study, ICAS in the ACA and PCA was linked to a higher risk of dementia and mild cognitive impairment, respectively (11).

The Stenting vs. Aggressive Medical Management for Preventing Recurrent Stroke in Intracranial Stenosis (SAMMPRIS), Vitesse Intracranial Stent Study for Ischemic Stroke Therapy (VISSIT), and China Angioplasty and Stenting for Symptomatic Intracranial Severe Stenosis (CASSISS) trials for sICAS found no superiority for intracranial stenting over aggressive medical management in preventing recurrent strokes (12–14). Aggressive medical management remains the primary therapeutic approach, while balloon angioplasty, stent placement, or both are considered as an alternative treatment for patients refractory to aggressive medical management (1, 15). In 2024, the Balloon Angioplasty for Symptomatic Intracranial Artery Stenosis (BASIS) trial implies that, for the first time, submaximal balloon angioplasty plus aggressive medical management outperforms aggressive medical management alone in terms of secondary stroke prevention in patients with sICAS (2). However, the internal carotid, middle cerebral, vertebral, or basilar arteries qualify for inclusion in the aforementioned four trials. Not included are the anterior and posterior cerebral arteries. In the real world, there exist but a limited number of recorded cases regarding endovascular procedures for sICAS associated with ACA or PCA that are refractory to medical therapy (16–24).

Therefore, this single-center, retrospective study aims to present our experience with percutaneous transluminal angioplasty and stenting (PTAS) in patients suffering from medically refractory sICAS associated with either ACA or PCA.

## 2 Materials and methods

### 2.1 Study design

This is a single-center, small-sample, retrospective study conducted with the authority of the Beijing Geriatric Hospital Ethics Committee (BJLNYY-2021-011). All patients were explicitly informed regarding the off-label application of the Neuroform stent system and provided signed informed consent.

### 2.2 Patients

From January 2022 to December 2024, 12 patients with medically refractory sICAS associated with ACA or PCA, who eventually received PTAS, were included for analysis. Exclusion criteria included (1) non-atherosclerotic stenosis, concomitant infarcts in other vascular territories, antiplatelet contraindication, a baseline modified Rankin Scale (mRS) score of 3 or above, and (2) concomitant disease with survival <2 years (24). The patient demographic data, clinical chart, indications for stent application, lesion features, procedural note, and post-procedural hospital course were reviewed.

sICAS refers to a recent transient ischemic attack (TIA) or ischemic stroke due to 70–99% atherosclerotic stenosis of a major intracranial artery (70–99% as per the Warfarin Aspirin Symptomatic Intracranial Disease method) (2, 25). Diffusion-weighted imaging on magnetic resonance imaging or a repeat head computed tomography scan verified an ischemic stroke. The extent of stenosis was assessed by digital subtraction angiography (DSA). sICAS with 70–99% stenosis and 2 or more strokes in the same vascular territory, with at least 1 stroke occurring during medical therapy, is considered refractory to medical treatment (26). Aggressive medical management, as suggested, entails dual antiplatelet therapy for up to 90 days, control of vascular risk factors with established targets, physical activity as tolerated, and smoking cessation (1). In this study, the preferred regimen for dual antiplatelet therapy was aspirin 100 mg daily and clopidogrel 75 mg daily. In cases of clopidogrel resistance, clopidogrel was substituted with ticagrelor at a dosage of 90 mg twice a day (1, 2).

### 2.3 Endovascular treatment

All patients underwent PTAS conducted by the same certified operator under general anesthesia via either the transfemoral or transradial access. Dual antiplatelet therapy was administered for at least 5 days prior to PTAS.

A vascular stenosis treatment that worked was marked by improved symptoms (as measured by the modified Rankin score), a decrease in the stenosis to below 30% (21), and no further ischemic strokes in the target vessel territory during the follow-up period. Procedural complications encompassed vasospasm, arterial dissection, pseudoaneurysm, arterial occlusion, arterial perforation, arterial rupture, hemorrhage, and thrombosis (2).

#### 2.3.1 For patients with ACA lesions

All three patients with A2 lesions underwent PTAS via the transfemoral access. An 8F Tracline™ access system (HEMO, Weihai, Shandong, China) was placed at the vertical part of the C4 segment of the internal carotid artery, accompanied by a 5F intermediate catheter (Ton-Bridge Medical, Zhuhai, Guangzhou, China). The 5F intermediate catheter was placed at the C6 segment (above the level of the ophthalmic artery).

After the intermediate catheter was in place, the stenotic lesion was traversed with a Synchro SELECT Standard Straight microwire (0.014 in * 300 cm; Stryker, Kalamazoo, MI, USA) guided by an optimal roadmap. Predilation was subsequently conducted using a moderately undersized, rapid exchangeable balloon at the nominal pressure (defined as a balloon inflation diameter 50–80% of the proximal artery diameter) (27, 28). (Achieva Medical, Shanghai, China). After angioplasty, the balloon catheter was exchanged for either a NOVA intracranial sirolimus-eluting stent system (SINOMED, Tianjin, China) or a microcatheter (Excelsior SL-10®, Stryker Neurovascular, Fremont, CA, USA) to deploy the self-expanding stent (Neuroform Atlas stent system, Stryker Neurovascular, Fremont, CA, USA). The NOVA intracranial sirolimus-eluting stent system was deployed at the nominal pressure as well.

#### 2.3.2 For patients with PCA lesions

Seven patients with PCA lesions underwent PTAS via the transfemoral access. A 6F NeuronMAX long sheath (Penumbra, Alameda, CA, USA) or a 6F MPD guiding catheter (Cordis, Miami Lakes, Miami, FL, USA) was placed at the V2 segment of the vertebral artery, accompanied by a 5F intermediate catheter (Ton-Bridge Medical, Zhuhai, Guangzhou, China, or Achieva Medical, Shanghai, China). The 5F intermediate catheter was placed at the V4 segment.

Two patients with PCA lesions underwent PTAS via the transradial access. A 5F intermediate catheter (Ton-Bridge Medical, Zhuhai, Guangzhou, China, or HEMO, Weihai, Shandong, China) was placed at the V4 segment guided by an angiographic catheter. The angiographic catheter’s tip can be adjusted in terms of curvature (Weiqiang Medical, Hangzhou, Zhejiang, China).

After the intermediate catheter was in place, the stenotic lesion was traversed with a Synchro SELECT Standard Straight microwire (0.014 in * 300 cm; Stryker, Kalamazoo, MI, USA) guided by an optimal roadmap. Predilation was subsequently conducted using a moderately undersized, rapid exchangeable balloon at the nominal pressure (Achieva Medical, Shanghai, China). After angioplasty, the balloon catheter was exchanged for a microcatheter (Excelsior SL-10® or XT-27™, Stryker Neurovascular, Fremont, CA, USA) to deploy the self-expanding stent (Neuroform Atlas stent system, Stryker Neurovascular, Fremont, CA, USA, or Neuroform EZ stent system, Stryker, Kalamazoo, MI, USA).

### 2.4 Postoperative management

A routine head computerized tomography scan was performed postoperatively. The dual antiplatelet medication postoperatively lasted for 90 days (employing the NOVA intracranial sirolimus-eluting stent system for 180 days) and then aspirin alone. Furthermore, patients’ specific vascular risk factors were addressed accordingly.

### 2.5 Imaging follow-up for in-stent restenosis

Patients received DSA or head and neck computed tomography angiography (CTA) post-PTAS to assess in-stent restenosis (ISR). ISR is defined as greater than 50% stenosis within or immediately adjacent (within 5 mm) of the implanted stent and >20% absolute luminal loss (29).

### 2.6 Statistical analysis

Due to the limited sample size in this study, 95% confidence intervals (CI) for outcomes such as ISR rate, mRS improvement rate, and stroke recurrence rate were calculated by R (version 4.3.1) with the Clopper-Pearson method.

## 3 Results

Table 1 shows the clinical characteristics and follow-up results of 12 patients with medically refractory sICAS associated with ACA or PCA who ultimately underwent PTAS. Table 2 delineates the characteristics of the stenotic lesions and the PTAS.

**Table 1:** The clinical features and follow-up results of 12 patients.

No.	Age/Sex	Vascular risk factors	Ischemic event	DAT	Stenotic location	mRS^1^	mRS^2^	Last follow-up	
								Stroke post-PTAS	ISR/Time/Method
1	76y/M	HT/DM/HP	2nd infarct	A + C	A2	1	0	–	– 6mo by DSA
2	61y/F	HT/DM/HP	2nd infarct	A + C	A2	2	1	–	– 12mo by DSA
3	71y/F	HP	2nd infarct	A + C	A2	2	3	3rd infarct at 2mo	– 2mo by CTA
4	61y/M	HT/DM/HP	TIA/infarct	A + C	P2	2	0	–	– 7mo by DSA
5	60y/F	HT/DM/HP/HCY	2nd infarct	A + C	P1	2	0	–	– 7mo by DSA
6	53y/M	HP/Smoking	2nd infarct	A + C	P2	1	0	–	– 11mo by CTA
7	69y/M	DM/HP/Smoking	TIA/infarct	A + C	P2	1	0	–	– 10mo by CTA
8	56y/F	HT/DM/HP	TIA/infarct	A + C	P2	1	0	–	– 8mo by DSA
9	65y/F	HT/HP	TIA/infarct	A + C	P2	2	0	–	– 8mo by DSA
10	59y/M	DM/HP/HCY/Smoking	TIA/infarct	A + C	P1	1	0	–	– 18mo by DSA
11	58y/M	HT/DM/HP/Smoking	TIA/infarct	A + C	P1	1	0	–	– 11mo by CTA
12	79y/M	DM/HP	TIA/infarct	A + T	P2	1	0	–	– 8mo by CTA

HT, hypertension; DM, diabetes mellitus; HP, hyperlipidemia; HCY, hyperhomocysteinemia; DAT, dual antiplatelet therapy; A + C, aspirin plus clopidogrel; A + T, aspirin plus ticagrelor; A2, the A2 segment of the anterior cerebral artery; P1 or P2, the P1 or P2 segment of the posterior cerebral artery; mRS^1^, mRS pre-PTAS; mRS^2^, mRS three months post-PTAS; mo, month or months; ISR, in-stent restenosis; DSA, digital subtraction angiography; CTA, computed tomography angiography; 2nd, a second time; 3rd, a third time; −, no stroke or no ISR.

**Table 2:** The features of stenotic lesions and PTAS of 12 patients.

No.	Stenotic location	Onset to PTAS/day^#^	PTAS access	Vessel size/mm		Lesion length/mm	Degree of stenosis	Balloon size/mm	Stent size/mm	Residual stenosis	Complications
				Proximal	Distal						
1	A2	15	TFA	2.06	1.80	7.31	95%	1.5*9	3.0*15^a^	28%	/
2	A2	15	TFA	2.20	2.11	8.07	95%	1.5*10	2.25*12^b^	17%	dissection
3	A2	14	TFA	2.10	1.84	11.24	93%	1.5*12	4.0*21^a^	22%	/
4	P2	16	TFA	2.24	2.24	5.78	78%	1.5*9	3.0*15^a^	8%	/
5	P1	15	TFA	1.96	1.96	6.76	79%	1.5*9	4.0*15^a^	25%	/
6	P2	17	TFA	2.09	1.89	5.91	78%	1.5*9	2.5*15^c^	<5%	/
7	P2	14	TRA	1.78	1.78	3.09	73%	1.5*9	3.0*15^a^	<5%	/
8	P2	15	TFA	1.97	1.78	4.36	80%	1.5*9	3.0*15^a^	<5%	/
9	P2	15	TFA	2.22	2.45^$^	4.79	90%	1.5*9	3.0*15^a^	7%	/
10	P1	14	TFA	2.28	1.95	10.81	84%	2.0*12	3.0*21^a^	10%	/
11	P1	25	TFA	2.15	2.25^$^	5.08	75%	2.0*9	4.0*21^a^	<5%	/
12	P2	27	TRA	1.78	1.89^$^	4.73	75%	1.5*9	4.0*15^a^	14%	/

PTAS, percutaneous transluminal angioplasty and stenting; TFA, transfemoral approach; TRA, transradial approach; ^#^days from the onset of recurrent ischemic event to PTAS; ^a^Neuroform Atlas stent system; ^b^NOVA intracranial sirolimus-eluting stent system; ^c^Neuroform EZ stent system; /, no complications; ^$^vessel expansion after the stenosis.

### 3.1 Patient characteristics

Among 12 patients, there were 7 males and 5 females, with ages ranging from 53 to 79 years and an average age of 64. All patients possessed vascular risk factors, including hypertension (7/12), diabetes mellitus (9/12), hyperlipidemia (12/12), hyperhomocysteinemia (2/12), and smoking (4/12). Regarding the medically refractory sICAS, 5 patients suffered infarction on two occasions, whereas 7 patients originally experienced a TIA that later progressed to infarction. One patient showed resistance to clopidogrel and received dual antiplatelet treatment with aspirin and ticagrelor. Three severe stenosis lesions were identified in the A2 segment of the ACA, while 9 lesions were found in the P1 or P2 segments of the PCA (stenosis > 70%).

### 3.2 Endovascular procedure

The interval from the onset of recurrent ischemic events to PTAS varied from 14 to 27 days, with a median interval of 15 days.

Two patients underwent procedures through transradial access, while 10 patients utilized transfemoral access. Stent deployment was technically successful in all patients. One patient utilized the NOVA intracranial sirolimus-eluting stent system, 1 patient employed the Neuroform EZ stent system, and the remaining 10 patients used the Neuroform Atlas stent system. The maximum residual stenosis was 28% across all lesions after PTAS.

**Figure 1:**
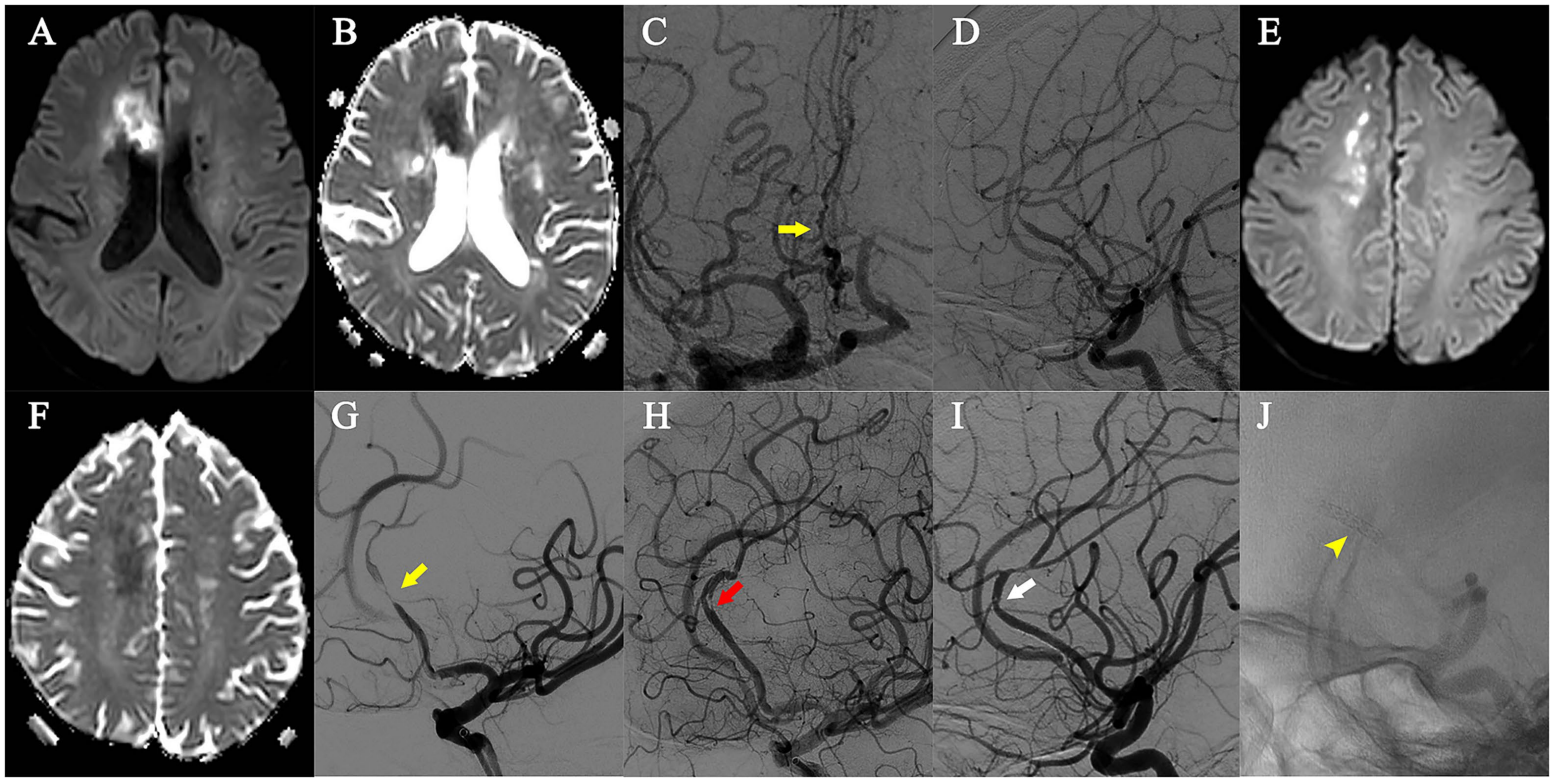
Patient No. 2. **(A,B)** Diffusion Weighted Imaging and Apparent Diffusion Coefficient indicated the first infarction in the right anterior cerebral artery territory. **(C,D)** During the first infarction, digital subtraction angiography showed the right anterior cerebral artery A2 segment stenosis (yellow arrow). **(E,F)** Diffusion Weighted Imaging and Apparent Diffusion Coefficient indicated the second infarction in the right anterior cerebral artery territory. **(G)** During the second infarction, digital subtraction angiography showed the right anterior cerebral artery A2 segment stenosis (yellow arrow). **(H)** Post-PTAS digital subtraction angiography showing dissection with no limited flow (red arrow). **(I)** Digital subtraction angiography 12 months after PTAS, dissection disappeared (white arrow), and in-stent restenosis was absent. **(J)** The implanted NOVA intracranial sirolimus-eluting stent (yellow arrowhead).

Dissection with no limited flow occurred in 1 patient (No. 2) during the NOVA stent implantation (Figure 1), while the remaining patients experienced no complications during or after the procedure.

### 3.3 Follow up

In the follow-up, 1 patient suffered a recurrent stroke (8.3, 95% CI: 0.21–38.5%) due to the cessation of antiplatelet and anticoagulant medication 2 months after endovascular stenting; however, the remaining 11 patients exhibited a fair outcome with a modified Rankin Scale of 1 or 0 (91.67, 95% CI: 61.5–99.8%) and no in-stent stenosis (0, 95% CI: 0–26.5%).

Patient No. 3 had a third infarction in the treated ACA territory 2 months later. She discontinued the dual antiplatelet therapy for anticoagulation due to deep venous thrombosis in the lower extremities. Subsequently, subcutaneous hemorrhage led to the termination of anticoagulation therapy. A third ischemic event occurred in the absence of antiplatelet or anticoagulant therapy, worsening her dysfunction. The CTA did not detect obvious ISR at the time of a third infarction.

**Figure 2:**
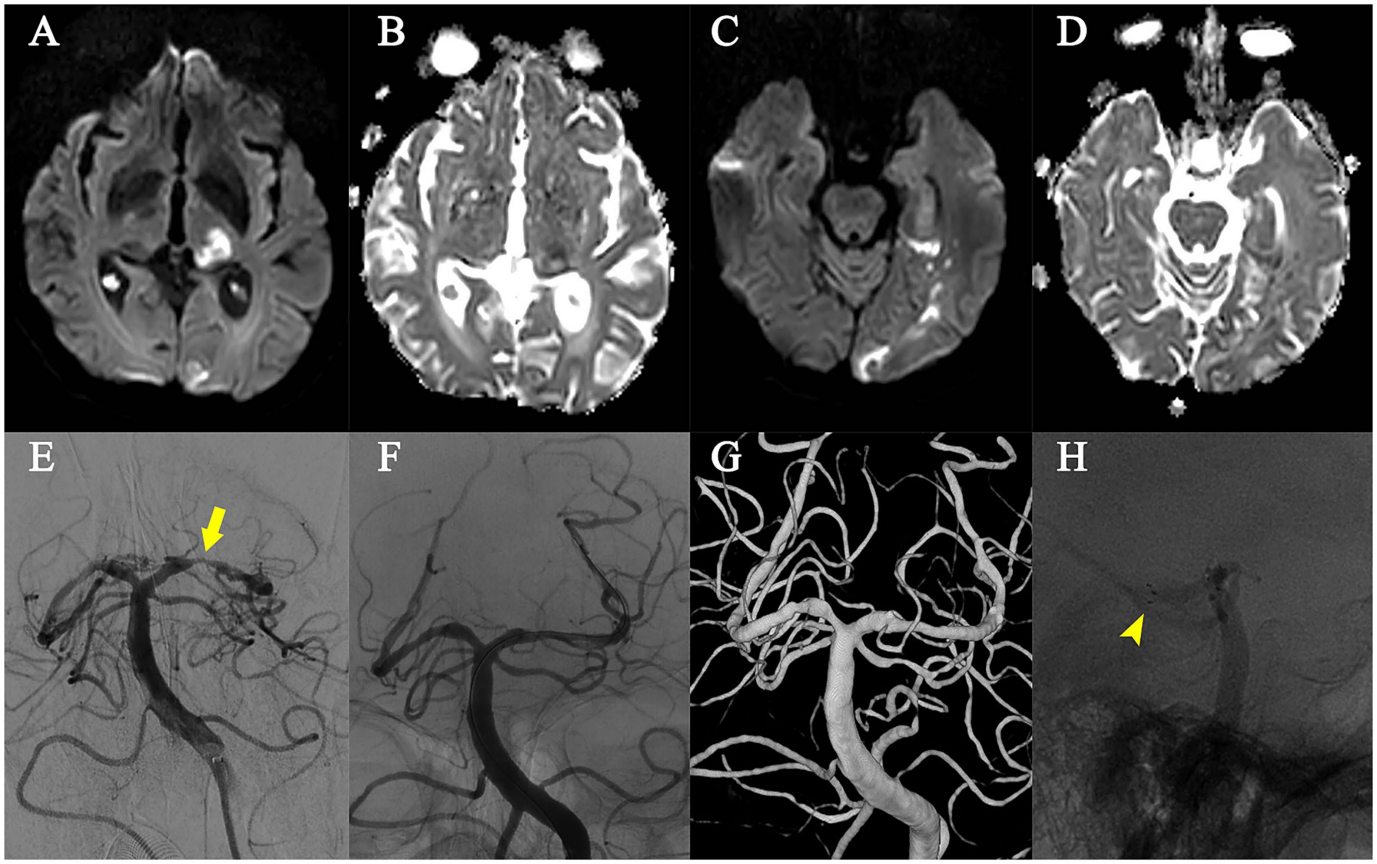
Patient No. 10. **(A–D)** Diffusion Weighted Imaging and Apparent Diffusion Coefficient indicated the two ischemic events (infarction) in the left posterior cerebral artery territory. **(E)** Digital subtraction angiography revealed the left posterior cerebral artery P2 segment stenosis (yellow arrow) during the infarction. **(F)** Post-PTAS digital subtraction angiography demonstrated the alleviated stenosis of the left posterior cerebral artery P2 segment. **(G)** Digital subtraction angiography 18 months after PTAS showed no in-stent restenosis. **(H)** The implanted Neuroform Atlas stent (yellow arrowhead).

No strokes were observed in the remaining 11 patients throughout the follow-up period of 6–18 months (mean time 9.6 months), and symptoms ameliorated at 3 months post-PTAS (modified Rankin Scale of 1 or 0). No ISR was detected by DSA or CTA in the last follow-up. (As shown in Figure 2). The dissection disappeared 12 months after PTAS in Patient No. 2 (Figure 1).

## 4 Discussion

To our knowledge, this is the highest number of cases concerning endovascular stenting for sICAS associated with ACA or PCA reported at a single center. Most patients experienced no periprocedural complications, achieved a reduction in stenosis to below 30%, showed improved symptoms, and did not suffer subsequent strokes or ISR during the follow-up period. This retrospective analysis suggests that endovascular stenting for patients with medically refractory sICAS associated with ACA or PCA is feasible and procedurally safe.

Anterior cerebral artery territory infarction is a rare phenomenon, representing 0.47–1.8% of all ischemic strokes [0.47% reported in Japan (6); 1.1% in Korea (5); 1.3% in Turkey (30) and Spain (31); 1.8% in Switzerland (32)]. Although posterior cerebral artery stroke occurs at a higher rate than ACA infarction, it still comprise no more than 5–10% of all ischemic strokes (33, 34). The small diameter of vessels, along with the low incidence of infarction, poses a significant obstacle to the progression of endovascular treatment in sICAS associated with ACA or PCA, especially given that the Neuroform stent system is off-label for ICAS and Wingspan is off-label for vessels under 2 mm (21). Previous case series utilizing the Neuroform or Wingspan stent system for treating sICAS associated with ACA or PCA demonstrated a 100% technical success rate without any complications (21, 24), aligning with the findings of this study.

The progression of endovascular technologies has facilitated the navigation and deployment of intracranial stents via microcatheters with increased efficiency and the potential for a safer intervention (35). In this study, the majority (11/12) of the deployed stents were from the Neuroform stent system, with a high rate of technical success and no periprocedural complications. The choice of stent was primarily influenced by the fact that the ACA and PCA are distal cerebral vessels and that the path of endovascular therapy is highly tortuous. The rigidity of the stent system links drug-eluting stents to a notable rate of unsuccessful placements (36). During stent delivery, the rigidity could lead to dissection or even rupture of the access vessel. The application of balloon-mounted stents, particularly in diminutive cerebral arteries, has heightened the risk of complications and poor durability for angioplasty (21, 37). In this study, one patient utilized the NOVA intracranial sirolimus-eluting stent system, and dissection with no restricted flow occurred. The reason for using a drug-eluting stent was that in this patient, the entire delivery path was relatively straight, facilitating the intermediate catheter to be smoothly advanced to the target site, and the angle between the ACA and the internal carotid artery was wider, allowing for unimpeded stent passage. Additionally, the diameters of the proximal and distal ends of the lesion vessel were suitable for the smallest balloon-mounted stent. The initial purpose of this attempt was to compare the outcomes in mid- to long-term follow-up between drug-eluting stents and bare-metal stents via microcatheters, given that the NOVA intracranial sirolimus-eluting stent system reduces the risks of ISR compared with bare-metal stents (37).

However, the newer self-expanding stents provide significant advantages in terms of safety and operational convenience. Most patients were treated with the smallest diameter stents, which were necessary because the normal arteries at both ends of the lesion were narrow. The Neuroform Atlas stent was primarily used due to its extensive application for aneurysms located in the middle and distal cerebral arteries. Although we did not measure the expansion diameter and deployed length post-stent implantation, our findings indicate that the stent can be successfully released and expanded in vessels with a diameter of 2 mm or less. Furthermore, after appropriate pre-stent balloon dilation, the stent implantation did not disrupt forward blood flow, with residual stenosis less than 30%. Therefore, it is suggestive that the newer self-expanding stent allows access and the ability to safely treat lesions in distal cerebral vessels.

The timing of the endovascular treatment is highly crucial. Premature endovascular treatment may result in an elevated risk of periprocedural complications. A delay in endovascular treatment may result in the loss of a therapeutic window (2). A pooled analysis of SAMMPRIS and VISSIT trials did not show any increased risk of 1 month stroke and/or death in patients who underwent intracranial stent placement within 7 days compared with those treated more than 7 days after the qualifying cerebral ischemic event (38). However, the lowest rate of 1-month stroke or all-cause death in the BASIS trial compared to the SAMPPRIS, VISSIT, and CASSISS trials may be partially owed to the appropriate median interval between symptom onset and patient enrollment with ischemic stroke. The median interval between symptom onset and patient enrollment with ischemic stroke in the BASIS trial was beyond 14 (34 [20–51]) days, which was longer than that in the SAMPPRIS (7 [4–16] days) and VISSIT (9 [0–42] days) trials and shorter than that in the CASSISS trial (38 [27–75] days) (2, 12–14). A previous single-center study showed that treatment of symptomatic small-artery (diameter ≤ 2 mm) ICAS with angioplasty and/or stenting was safe and effective, where the mean time from initial symptom to interventional procedure was 4.8 months (24). In this study, the time between the initiation of recurrent ischemic events and PTAS was between 14 and 27 days (median: 15 days), potentially aiding in the reduction of complications.

Perforator occlusion is the predominant periprocedural complication observed (39), believed to arise from a “snowplowing effect” (balloon angioplasty or stenting mechanically displacing the atherosclerotic plaque into the ostia of side branches, thus occluding side branches) (40). Unlike the BASIS trial, which recommends submaximal balloon angioplasty with a dedicated intracranial balloon without stent implantation, all patients in the present study underwent PTAS. No perforator occlusion occurred in this study and the previous case series that used the Neuroform or Wingspan stent system (21, 24). One main reason may be that, in comparison to the middle cerebral artery and basilar artery, the anterior and posterior cerebral arteries possess fewer perforating branches. Another reason is that this study used predilation with a moderately undersized balloon to achieve submaximal balloon angioplasty. The last one may owe to the reduced radial support force and a large mesh size of the Neuroform stent system, which reduces the likelihood of damage to small branches (41). Nonetheless, owing to the limited sample size of this study, it is essential to gather more cases to ascertain the influence of this operation on the perforating branches, particularly the P1 segment, which possesses more perforating branches.

A significant challenge in stenting is restenosis, which is associated with a high incidence of recurrent stroke (42). A prior study on small-artery ICAS reported that only 1 patient (1/10) had restenosis, which occurred 1 month after primary angioplasty. In other small-artery ICAS publications, the overall restenosis rate for primary angioplasty was 40.0 and 27.7% for PTAS (24). In a meta-analysis of large-artery ICAS literature, a comparison of primary angioplasty versus PTAS treatment showed restenosis rates (14.2 vs. 11.1%, *p* = 0.04) were significantly lower in the PTAS group (43). Low residual stenosis could increase in-stent restenosis for symptomatic atherosclerotic stenosis (44). PTAS, submaximal balloon angioplasty, and the Neuroform stent system with reduced radial support force all may help prevent ISR in this study. Since CTA is less effective than DSA for detecting restenosis and the follow-up period was short, further investigation into the occurrence of ISR with DSA is necessary.

The path of endovascular therapy in the ACA is more complex than that of the PCA, influenced by the internal carotid artery siphon, the angle between the terminus of the internal carotid artery and the A1 segment, and the relationship between the A1 and A2 segments. Consequently, the microwire necessitates better shaping and meticulous operation, which requires a more experienced operator. This complexity results in an extended operation time, which correlates with an increased likelihood of embolism (15). In this study, two patients with PCA lesions underwent PTAS via the transradial access. A previous study suggested that using the transradial access significantly shortens the endovascular therapy duration for the posterior circulation compared to the transfemoral access (45). Simultaneously, the transradial access eliminates the necessity to operate within the aortic arch, potentially reducing the risk of embolic events associated with possible trauma to the aortic wall (46). Therefore, endovascular therapy for PCA using the transradial access offers specific advantages to some degree. Another point to note is that the endovascular therapy for PCA requires passing through the perforator-bearing basilar artery. During the advancement of the microwire, care must be taken to avoid damaging the perforators (47).

This research has several limitations. First, this study is a small-sample, single-center, retrospective analysis, and patient selection or treatment indication may have been influenced by operator discretion. Second, most trials to date have focused on high-grade (>70%) stenosis. This study is the same. While the severity of stenosis is one of the predictors of stroke in both symptomatic and asymptomatic patients with ICAS, high-resolution magnetic resonance imaging can improve patient selection and device efficacy to further improve the safety of endovascular intervention (15). Meanwhile, patients most likely to benefit from the endovascular interventions are those with hemodynamic compromise (35). This study did not utilize perfusion imaging (e.g., arterial spin labeling, computed tomography or magnetic resonance perfusion imaging), which is a notable limitation that could affect the identification of high-risk hemodynamic states. Finally, the short follow-up duration of this study may be insufficient to comprehensively evaluate the occurrence of ISR or long-term ischemia recurrence. Long-term follow-up is necessary for these patients.

Endovascular stenting with newer self-expanding stents may be feasible and procedurally safe for patients with medically refractory sICAS associated with ACA or PCA, but it is highly dependent on the skill and experience of the operator. In terms of technical success, it involves the high positioning of the intermediate catheter, the use of an undersized balloon for submaximal balloon angioplasty, the implementation of the smaller-diameter stents through microcatheter, comprehensive perioperative preparation, and highly selected patients. However, we must state that this analysis is a small-sample exploratory study. This kind of endovascular treatment for now should not be treated as a generalizable, low-risk therapy and is not recommended for low-volume centers or operators lacking experience in managing ICAS with PTAS. Further research will be necessary to guarantee that the benefits outweigh any possible complications in a larger patient population.

## Data Availability

The raw data supporting the conclusions of this article will be made available by the authors, without undue reservation.
